# Bidirectional cross-regulation between the endothelial nitric oxide synthase and β-catenin signalling pathways

**DOI:** 10.1093/cvr/cvu173

**Published:** 2014-07-25

**Authors:** Christina M. Warboys, Nan Chen, Qiuping Zhang, Yasin Shaifta, Genevieve Vanderslott, Gabriella Passacquale, Yanhua Hu, Qingbo Xu, Jeremy P.T. Ward, Albert Ferro

**Affiliations:** 1 Cardiovascular Division, Department of Clinical Pharmacology, British Heart Foundation Centre of Research Excellence, King's College London, 3.07 Franklin-Wilkins Building, 150 Stamford Street, London, UK; 2 Division of Asthma, Allergy, and Lung Biology, King's College London, London,UK

**Keywords:** Nitric oxide, Beta catenin, Gene transcription, Angiogenesis

## Abstract

**Aims:**

β-catenin has been shown to be regulated by inducible nitric oxide synthase (NOS) in endothelial cells. We investigated here whether β-catenin interacts with and regulates endothelial NOS (eNOS) and whether eNOS activation promotes β-catenin signalling.

**Methods and results:**

We identified β-catenin as a novel eNOS binding protein in human umbilical vein endothelial cells (HUVECs) by mass spectroscopy and western blot analyses of β-catenin and eNOS immunoprecipitates. This was confirmed by *in situ* proximity ligation assay. eNOS activity, assessed by cGMP production and eNOS phosphorylation (Ser1177), was enhanced in β-catenin^−/−^ mouse pulmonary endothelial cells (MPECs) relative to wild-type MPECs. eNOS activation (using adenosine, salbutamol, thrombin, or histamine), or application of an NO donor (spermine NONOate) or cGMP-analogue (8-bromo-cGMP) caused nuclear translocation of β-catenin in HUVEC as shown by western blotting of nuclear extracts. Exposure to spermine NONOate, 8-bromo-cGMP, or sildenafil (a phosphodiesterase type 5 inhibitor) also increased the expression of β-catenin-dependent transcripts, IL-8, and cyclin D1. Stimulation of wild-type MPECs with basic fibroblast growth factor (bFGF), vascular endothelial growth factor (VEGF), spermine NONOate, 8-bromo-cGMP, or sildenafil increased tube length relative to controls in an angiogenesis assay. These responses were abrogated in β-catenin^−/−^ MPECs, with the exception of that to bFGF which is NO-independent. In C57BL/6 mice, subcutaneous VEGF-supplemented Matrigel plugs containing β-catenin^−/−^ MPECs exhibited reduced angiogenesis compared with plugs containing wild-type MPECs. Angiogenesis was not altered in bFGF-supplemented Matrigel.

**Conclusion:**

These data reveal bidirectional cross-talk and regulation between the NO-cGMP and β-catenin signalling pathways.

## 1. Introduction

Nitric oxide (NO), generated by the vascular endothelium through the action of endothelial NO synthase (eNOS), plays an essential part in vascular homeostasis, principally through causing vasodilation and inhibiting platelet function, thrombogenesis, vascular smooth muscle cell proliferation, and progression of atherosclerosis.^1,2^ Classically, eNOS is activated through an increase in cytosolic Ca^2+^ and consequent binding of Ca^2+^-calmodulin.^3^ However, it is now apparent that activation can also occur independently of any rise in cytosolic Ca^2+^, via phosphorylation on specific residues and/or association with proteins such as β-actin,^4^ heat shock protein-90, caveolin-1, and dynamin-2, as previously reviewed.^5^

Recently, inducible NOS (iNOS) has been demonstrated to regulate the function of β-catenin, a key component of the endothelial adherens junction, in endothelial cells.^6^ Furthermore, in MLO-Y4 osteocytes, NO has been shown to activate the β-catenin signalling pathway;^7^ and a recent study reported that NO increases endothelial permeability through *S*-nitrosylation of β-catenin in response to vascular endothelial growth factor (VEGF).^8^

Aside from its importance in the regulation of permeability, β-catenin in concert with T-cell factor/lymphocyte enhancing factor (TCF/LEF) transcription factors, also plays an important role in transcriptional regulation of various genes and is a key modulator in the Wnt signalling pathway.^9^ β-catenin target genes include c-Myc^10^ cyclin D^11^ and axin-2,^12^ as well as the sox (sox-9, sox-17) and matrix metalloproteinase (MMP-2, MMP-9, MMP-7) families.^13–16^ NO has also long been known to regulate downstream gene expression. As examples, activity of the transcription factor c-jun is inhibited by NO,^17^ and NF-κB activity is down-regulated due to NO-induced *S*-nitrosylation.^18^ A potential link between transcriptional regulation by NO and β-catenin has been revealed in conditionally immortalized murine colonic epithelial cells, where the NO donor NOR-1 was reported to induce β-catenin association with LEF in the nucleus.^19^

We hypothesized that endothelium-derived NO may induce β-catenin to translocate from the cytosol and/or plasmalemma of endothelial cells to the nucleus, thereby inducing changes in gene transcription. The aims of this study were three-fold: first to investigate whether β-catenin interacts directly with eNOS in endothelial cells; secondly to ascertain whether such interaction modulates eNOS activity; and thirdly to determine whether activation of NO-cGMP signalling—either through endogenous eNOS stimulation or through other means—gives rise to nuclear translocation of β-catenin with resultant gene transcriptional and functional effects.

## 2. Methods

Detailed methods are available in the Supplementary material online.

### 2.1 Cell culture

Human umbilical vein endothelial cells (HUVECs) were isolated as previously described.^20^ Wild-type and β-catenin^−/−^ mouse pulmonary endothelial cells (MPECs) were a generous gift from Professor Elisabetta Dejana, University of Milan, Italy.^21^

### 2.2 Proximity ligation assay

HUVECs cultured in 16-well chamber slides (30 000 cells/well) were fixed with 4% paraformaldehyde then permeabilized with 0.5% TX-100. *In situ* proximity ligation assay (PLA) was carried out using rabbit anti-eNOS and mouse anti-β-catenin primary antibodies in combination with Duolink *In Situ* Red Detection Kit (Sigma). PLA was carried out according to the manufacturer's instructions.

### 2.3 Preparation of lysates

Cell lysates were prepared as previously described.^20^ For preparation of nuclear lysates, hypotonic buffer followed by centrifugation was used to collect the cytosolic fraction (supernatant). Pellets (containing the nuclei) were lysed with high-salt buffer.

### 2.4 Immunoprecipitation and western blotting

Immunoprecipitation of eNOS or β-catenin from 400 µg of cell lysate with 1 µg of antibody was carried out as previously described using Protein G Sepharose beads. Electrophoresis and electroblotting were performed as described.^4^

### 2.5 cGMP ELISA

MPEC lysates were extracted in the presence of 3-isobutyl-1-methylxanthine (IBMX; 1 mmol/L) by addition of 0.1 mol/L HCl. cGMP concentration was assessed following acetylation using Cyclic GMP EIA Kit (Cayman Chemicals) according to the manufacturer's instructions. Resulting cGMP concentrations were normalized to protein content per sample.

### 2.6 Angiogenesis assays

To evaluate angiogenesis (tube formation) *in vitro*, MPECs were seeded onto Matrigel; total tube length was assessed after 6 h. To investigate *in vivo* angiogenesis, Matrigel supplemented with either VEGF or basic fibroblast growth factor (bFGF) was mixed with MPECs loaded with Vybrant® DiO cell labelling solution (Life Technologies) and injected into C57BL/6 mice anaesthetized using isoflurane. Following sacrifice, plugs were excised, snap-frozen, and cryosectioned for immunostaining with an anti-CD31 antibody and imaged by confocal microscopy. Capillary formation was assessed by determining the percentage of CD31-positive Vybrant-labelled MPECs compared with the total number of CD31-positive cells within four randomly selected fields of view per plug. Animals were studied in accordance with Directive 2010/63/EU of the European Parliament.

### 2.7 Quantitative RT–PCR

RNA was isolated using QIAGEN RNeasy Kit according to the manufacturer's instructions. Transcript levels were assessed by quantitative real-time PCR using gene-specific primers.

### 2.8 Statistical analysis

All data are presented as mean ± SEM. Statistical analysis was performed using GraphPad Prism (Version 6.0) software. Unless indicated otherwise, all statistical comparisons were by one-way analysis of variance (ANOVA) with repeated measures and the Geisser-Greenhouse correction for sphericity. Fisher's LSD test was used for planned *post-hoc* pairwise comparisons. Two-way ANOVA was carried out where inhibitors were used in conjunction with agonists. Where responses of wild-type and β-catenin^−/−^ MPECs were examined, responses within each cell type were analysed separately by ANOVA as appropriate. In all cases, *P* < 0.05 (two-sided) was taken to indicate statistical significance.

## 3. Results

### 3.1 β-catenin associates with eNOS in HUVEC

**Figure 1:**
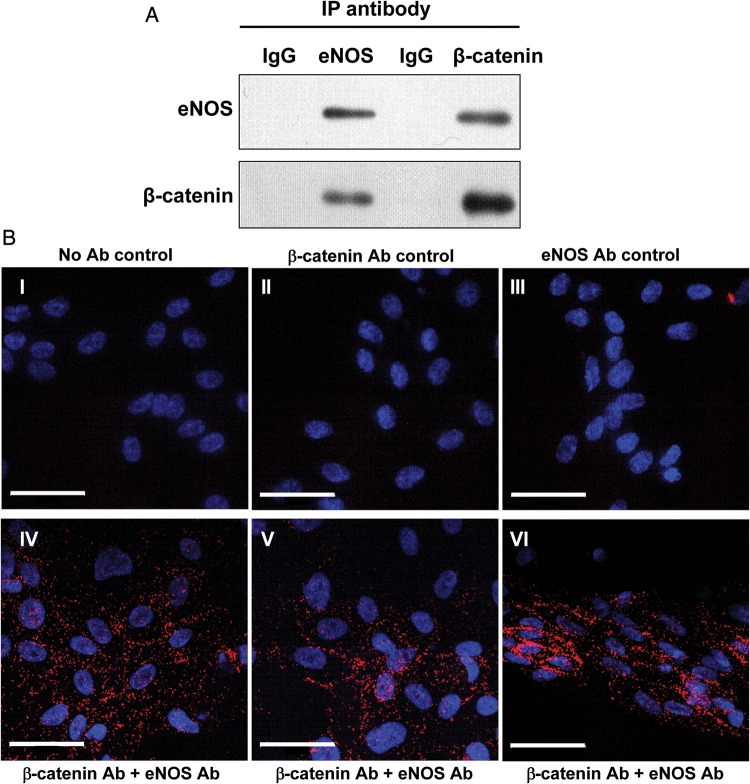
eNOS and β-catenin are associated in HUVECs. (*A*) HUVEC lysates were immunoprecipitated (IP) using either anti-eNOS or anti-β-catenin antibody, or corresponding control antibodies (mouse IgG or rabbit IgG, respectively), followed by western blotting to probe for either eNOS or β-catenin. IP of either protein resulted in detection of the other (experiment shown is representative of *n* = 3). (*B*) HUVECs were fixed and subject to PLA using antibodies targeting eNOS and β-catenin (images IV–VI). Amplification products are observed throughout the cells, particularly at cell borders. In control samples where PLA was carried out in the absence of antibodies (I), or β-catenin (II) or eNOS antibody (III) were used alone, no amplification products were observed. Scale bar shows 50 µm.

To investigate whether β-catenin associates with eNOS, eNOS was immunoprecipitated from HUVEC lysates, and the resulting immunoprecipitates were subjected to western blotting for both eNOS and β-catenin (*Figure 1A*). Immunoprecipitation of eNOS resulted in the detection of both eNOS and β-catenin, and similarly immunoprecipitation of β-catenin resulted in the detection of both proteins. The association between eNOS and β-catenin was confirmed *in situ* in fixed cells by PLA (*Figure 1B*). Amplification products were detected in HUVEC exposed to both eNOS and β-catenin antibodies, indicative of close proximity (<40 nm) of the two proteins; in contrast, controls where antibodies were omitted or used individually did not yield amplification products. Furthermore, peptide mass fingerprinting confirmed the presence of β-catenin in eNOS immunoprecipitates (Supplementary material online, *Figure S1*).

### 3.2 β-catenin regulates eNOS activity

**Figure 2:**
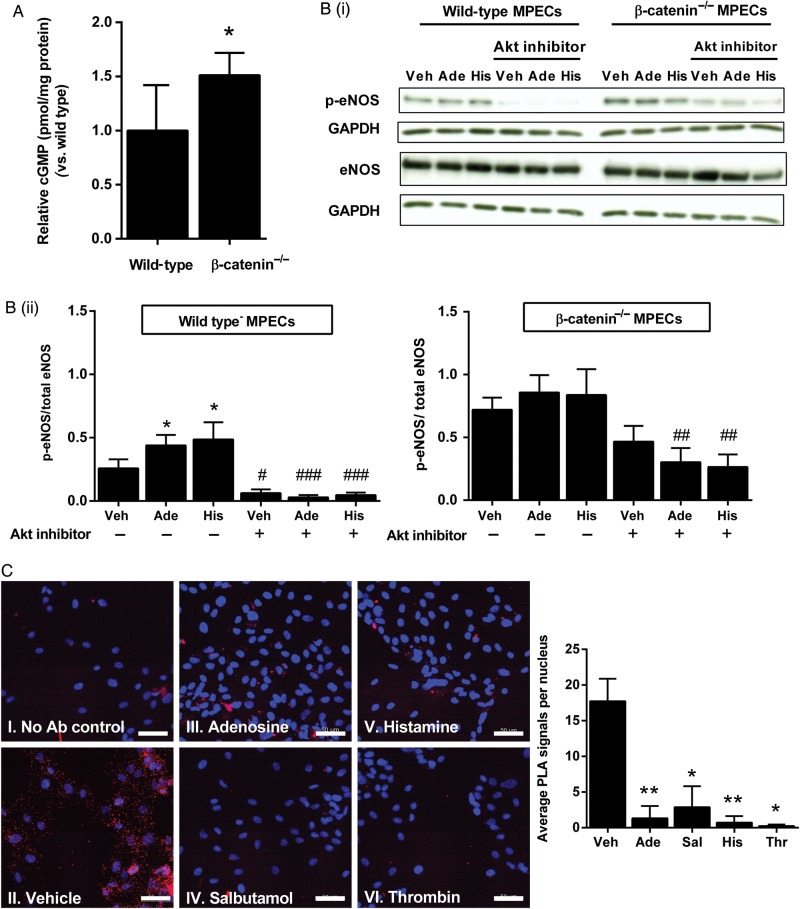
Loss of β-catenin increases basal eNOS phosphorylation and cGMP production. (*A*) cGMP production was assessed in wild-type and β-catenin^−/−^ MPECs by ELISA and results expressed relative to protein content per sample (shown relative to wild-type; *P < 0.05, unpaired *t*-test, *n* = 4 per group). (*B*) Wild-type and β-catenin^−/−^ MPECs were treated with adenosine (100 μmol/L), histamine (100 μmol/L), or vehicle for 2.5 min in the presence or absence of Akt inhibitor (10 μmol/L; pre-treatment for 30 min). *B*(i), Cell lysates were probed by western blotting for phosphoSer-1177-eNOS and total eNOS, and *B*(ii) results expressed as the densitometric ratio of phosphoSer-1177-eNOS/GAPDH to total eNOS/GAPDH (*P < 0.05 vs. vehicle, ^#^*P* < 0.05, ^###^*P* < 0.001 vs. corresponding treatment in the absence of Akt inhibitor; two-way ANOVA; *n* = 6 per group). Wild-type vehicle vs*.* β-catenin^−/−^ vehicle were analysed separately by unpaired *t*-test (**P < 0.01). (*C*) HUVECs were treated with adenosine (100 μmol/L), salbutamol (1 µmol/L), histamine (100 μmol/L), thrombin (1 IU/mL), or vehicle for 2.5 min then fixed and subject to PLA using antibodies targeting eNOS and β-catenin (II–IV). PLA was also carried out with the omission of primary antibodies (I). Images are representative of three independent cultures (scale bar shows 50 µm). Images were quantified using ImageJ Particle Analyser to determine the number of PLA signal per image and expressed as the mean number of signals per nucleus (*P < 0.05, **P < 0.01, one-way ANOVA with repeated measures, *n* = 3 per group).

To determine whether β-catenin binding to eNOS may serve to regulate eNOS function, we first measured intracellular cGMP, a well-validated index of bioactive NO, in wild-type and β-catenin^−/−^ MPECs. The absence of β-catenin protein in β-catenin^−/−^ MPECs was confirmed by western blotting (Supplementary material online, *Figure S2*). cGMP was higher in β-catenin^−/−^ compared with wild-type MPECs (*Figure 2A*). We next assessed eNOS phosphorylation status on Ser1177, a sensitive index of eNOS activity, by western blotting of cell lysates using an antibody that specifically recognizes phosphoSer-1177-modified eNOS; this was performed both on unstimulated and stimulated (with adenosine or histamine, each at 100 µmol/L for 2.5 min) wild-type and β-catenin^−/−^ MPECs. Stimulation with either agent increased eNOS phosphorylation in wild-type MPECs, and this was abrogated by pre-treatment with Akt inhibitor IV (10 µmol/L; Calbiochem). Moreover, we found that β-catenin^−/−^ MPECs exhibited significantly higher levels of eNOS phosphorylation under non-stimulated conditions, which were only partially reduced by the Akt inhibitor, and that stimulation of these cells with adenosine or histamine did not produce any further rise in eNOS phosphorylation (*Figure 2B*). These data suggest that β-catenin may function as an endogenous negative regulator of eNOS in endothelial cells.

### 3.3 Pharmacological activation of eNOS reduces association with β-catenin

Having demonstrated that β-catenin associates with, and tonically inhibits, eNOS activity in unstimulated cells we sought to determine, using PLA, whether the interaction is maintained following pharmacological activation of eNOS. Amplification products were observed throughout the cell and localized close to cell borders in vehicle-treated HUVECs; however, following stimulation for 2.5 min with adenosine (100 µmol/L), salbutamol (1 µmol/L), histamine (100 µmol/L), or thrombin (1 IU/mL), few amplification products were observed, suggesting a loss of interaction between eNOS and β-catenin following eNOS activation (*Figure 2C*).

### 3.4 Agents which activate eNOS and NO-cGMP induce nuclear translocation of β-catenin

**Figure 3:**
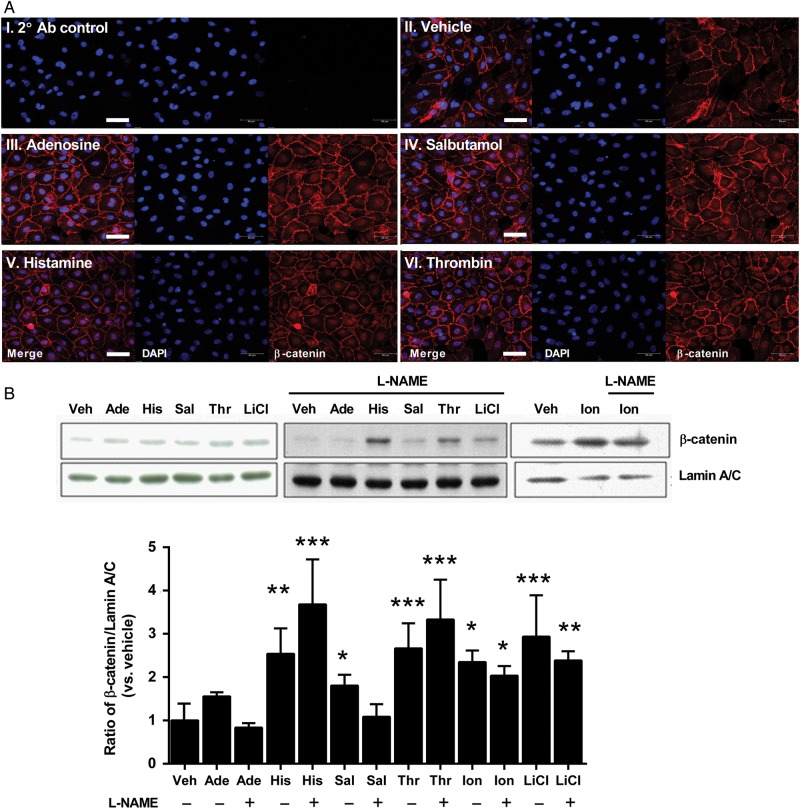
Activation of eNOS induces nuclear translocation of β-catenin in HUVECs. (*A*) HUVECs were treated with vehicle (II), 100 µmol/L adenosine (III), 1 µmol/L salbutamol (IV) 100 µmol/L histamine (V), or 1 IU/mL thrombin (VI) for 2.5 min, then fixed and immunostained for β-catenin (red). Cells were mounted in medium containing DAPI (blue) and imaged using a confocal microscope. Samples were included where primary antibody was omitted to control for non-specific binding of the secondary antibody (I). β-catenin was not observed in the nucleus of vehicle-treated cells, whereas all agonists elicited accumulation of β-catenin in the nuclear or peri-nuclear region. Images shown are representative of *n* = 4 experiments. (*B*) HUVECs were treated as above or with 10 µmol/L ionomycin or with 10 mmol/L LiCl, in the absence or presence of L-NAME (100 µmol/L), and nuclear extracts probed by western blotting for β-catenin. Results are expressed as the densitometric ratio of β-catenin to lamin A/C (shown relative to vehicle). *P < 0.05, **P < 0.01, ***P < 0.01 vs*.* vehicle (two-way ANOVA; *n* = 5–12 per group). Scale bar shows 50 µm.

We next determined whether eNOS stimulation or NO-cGMP pathway activation by other means induces β-catenin to translocate to the nucleus, an initial first step in its mediating effects on downstream gene transcription. HUVECs were treated with adenosine (100 µmol/L), salbutamol (1 µmol/L), histamine (100 µmol/L), or thrombin (1 IU/mL)—all at concentrations which we have previously found to cause maximal eNOS activation—or vehicle for 2.5 min, following which cells were fixed and immunostained for β-catenin, and imaged by confocal microscopy. In vehicle-treated cells, β-catenin was found mainly in the plasma membrane, in an even distribution, with minimal nuclear staining. In contrast, treatment with any of the eNOS agonists caused clear accumulation of β-catenin staining in the nuclear and/or peri-nuclear region (*Figure 3A*), with no obvious change in plasmalemmal staining.

To confirm that eNOS activation gives rise to accumulation of β-catenin within the nucleus, HUVECs were treated as above and nuclear proteins were extracted. Levels of nuclear β-catenin were determined by western blotting of nuclear extracts, with normalization to the nuclear membrane protein lamin A/C. All eNOS agonists tested induced an increase of nuclear β-catenin (*Figure 3B*). However, whereas the nuclear accumulation of β-catenin induced by adenosine and salbutamol (which do not elevate cytosolic Ca^2+^) was completely inhibited by the NOS inhibitor N^G^-nitro-L-arginine methyl ester (L-NAME; 100 µmol/L), that induced by histamine and thrombin (which activate eNOS by elevating cytosolic Ca^2+^) was not (*Figure 3B*). This suggests that both NO and an elevated cytosolic Ca^2+^ can independently induce translocation of β-catenin to the nucleus, the latter irrespective of eNOS activation. Consistent with this, translocation of β-catenin to the nucleus induced by the Ca^2+^ ionophore ionomycin (10 µmol/L) was similarly unaffected by the presence of L-NAME (*Figure 3B*). Glycogen synthase kinase (GSK)-3β phosphorylates β-catenin on Ser37 and Thr41, promoting its ubiquitination and degradation.^22^ We therefore also examined the effect of LiCl (which inhibits GSK-3β^23^) on nuclear accumulation of β-catenin, as a positive control. As expected, LiCl (10 mmol/L) increased nuclear β-catenin in HUVEC which was unaffected by co-incubation with L-NAME (*Figure 3B*).

We also examined whether eNOS, following its activation, co-translocates to the nucleus with β-catenin. Treatment with the Ca^2+^-elevating agonists, histamine and thrombin, induced nuclear translocation of eNOS (which was unaffected by co-incubation with L-NAME), whereas treatment with adenosine and salbutamol did not (Supplementary material online, *Figure S3*). These results show that, although β-catenin and eNOS co-associate in the plasma membrane, β-catenin translocation to the nucleus following eNOS activation (at least by agonists that do not raise intracellular Ca^2+^) does not require translocation of eNOS.

**Figure 4:**
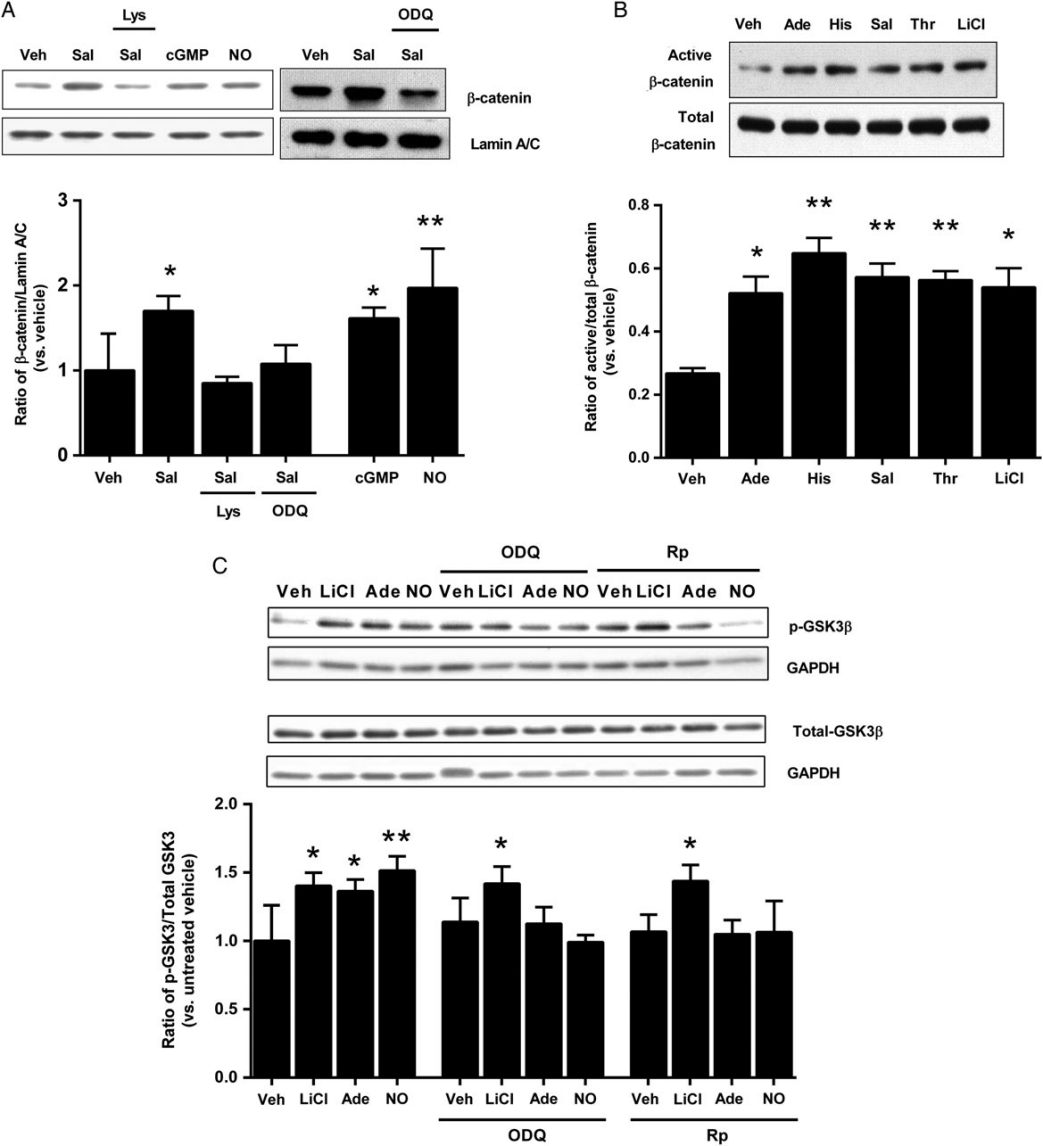
eNOS activation and NO-cGMP induce nuclear translocation and activation of β-catenin and inhibition of GSK-3β. (*A*) HUVECs were exposed to salbutamol for 2.5 min, either in the absence or presence of L-lysine (Lys; 1 mmol/L) or ODQ (10 µmol/L), or to 8-bromo-cGMP (cGMP; 10 μmol/L) or spermine NONOate (NO; 10 μmol/L) for 2.5 min. Nuclear extracts were subjected to western blotting for β-catenin and lamin A/C. Results are expressed as the densitometric ratio of β-catenin to lamin A/C, and shown relative to vehicle (*P < 0.05, **P < 0.01 vs*.* vehicle, one-way ANOVA without repeated measures, *n* = 5–8 per group). (*B*) Following exposure to adenosine (Ade; 100 µmol/L), histamine (His; 100 µmol/L), salbutamol (Sal; 1 µmol/L), thrombin (Thr; 1 IU/mL) or LiCl (10 mmol/L) for 2.5 min, whole cell lysates were probed by western blotting for active and total β-catenin. Results are expressed as the ratio of active to total β-catenin (*P < 0.05, **P < 0.01 vs*.* vehicle, one-way ANOVA with repeated measures; *n* = 4 per group). (*C*) HUVECs were exposed to LiCl (10 mmol/L), adenosine (Ade; 100 µmol/L) or spermine NONOate (NO; 10 µmol/L) for 2.5 min, in the presence or absence of ODQ (10 µmol/L) or Rp-8-bromo-guanosine 3′5′-cyclic monophosphorothioate (Rp; 10 µmol/L). Cell lysates were probed for total and phosphorylated GSK-3β. All results are expressed as the densitometric ratio of phosphorylated-GSK-3β/GAPDH to total GSK-3β/GAPDH and shown relative to vehicle (*P < 0.05, **P < 0.01 vs*.* vehicle, two-way ANOVA; *n* = 5 per group).

On this basis, we hypothesized that eNOS-mediated translocation of β-catenin to the nucleus was caused by NO production with consequent activation of soluble guanylyl cyclase (sGC) and cGMP generation. As elevation of cytosolic Ca^2+^ clearly activates a separate pathway (as shown earlier), we used salbutamol (which activates eNOS without elevating cytosolic Ca^2+23^) as the agonist. HUVECs were treated with salbutamol for 2.5 min, in the presence or absence of either L-lysine (1 mmol/L), which competes with L-arginine for transport into the cell and suppresses Ca^2+^-independent activation of eNOS,^23^ or 1H-[1,2,4]oxadiazolo[4,3-a]quinoxalin-1-one (ODQ, 10 µmol/L), a specific inhibitor of sGC. To directly determine the effects of cGMP and NO, we also stimulated the cells with the cell-permeable cGMP analogue, 8-bromo-cGMP (10 µmol/L), or the NO donor, spermine NONOate (10 µmol/L), for 2.5 min. Consistent with the hypothesis that NO and cGMP mediate translocation of β-catenin, the salbutamol-induced elevation of nuclear β-catenin was abolished by both L-lysine and ODQ, and both 8-bromo-cGMP and spermine NONOate induced elevation of nuclear β-catenin (*Figure 4A*).

### 3.5 eNOS activation increases levels of active (dephosphorylated) β-catenin

Nuclear translocation of β-catenin is typically associated with its activation, requiring dephosphorylation of Ser37 and Thr41. We examined activation of β-catenin using the clone 8E7 antibody (anti-ABC, Millipore), which recognizes active β-catenin dephosphorylated on Ser37 and Thr41.^24^ All four of the eNOS agonists tested induced an increase in active β-catenin in whole cell lysates, with no change in total β-catenin, as did LiCl which was used as a positive control (*Figure 4B*).

### 3.6 eNOS activation and NO inhibit GSK-3β by activating cGMP-dependent protein kinase

Having established a role for sGC activation and cGMP generation in inducing translocation of β-catenin to the nucleus, we reasoned that cGMP-dependent protein kinase (PKG) may be important in this process by stabilizing β-catenin through phosphorylation (and hence inhibition) of GSK-3β.^25^ HUVECs were treated with adenosine (a Ca^2+^-independent activator of eNOS^23^) or spermine NONOate for 2.5 min, in the presence or absence of either ODQ or the PKG inhibitor Rp-8-bromo-guanosine 3′5′-cyclic monophosphorothioate (Rp; 10 µmol/L). Phosphorylation of GSK-3β was assessed using an antibody that recognizes GSK-3β phosphorylated on Ser9 (Cell Signaling Technology). Adenosine and spermine NONOate both increased the ratio of phospho-GSK-3β/total GSK-3β, and these increases were abolished by either ODQ or Rp. In contrast, although LiCl increased the phospho-GSK-3β/total GSK-3β ratio, this was unaffected by either ODQ or Rp (*Figure 4C*).

### 3.7 NO-cGMP pathway activation increases expression of β-catenin target genes

**Figure 5:**
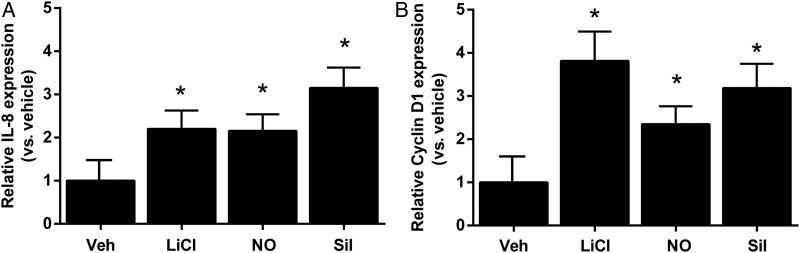
NO-cGMP signalling results in the induction of β-catenin-dependent target genes. HUVECs were treated with LiCl (10 mmol/L), spermine NONOate (NO; 10 µmol/L), or sildenafil (Sil; 100 nmol/L) for 12 h. Transcript levels of IL-8 (*A*) and cyclin D1 (*B*) were assessed by quantitative RT–PCR using HPRT as a housekeeping gene and quantified using the ΔΔCt method and shown relative to vehicle (*P < 0.05 vs. vehicle, one-way ANOVA with repeated measures; *n* = 4 per group for cyclin D1 and *n* = 5 per group for IL-8).

To ascertain whether nuclear translocation of β-catenin induced by the NO-sGC-cGMP pathway gives rise to transcriptional activation, we assessed the expression of cyclin D1^11^ and interleukin 8 (IL-8),^26,27^ which are β-catenin/TCF target genes involved in the regulation of angiogenesis, 12 h after stimulation with spermine NONOate or the phosphodiesterase inhibitor sildenafil (100 nmol/L). Both agents increased expression of IL-8 (*Figure 5A*) and cyclin D1 (*Figure 5B*) relative to vehicle, as did LiCl (included as a positive control).

### 3.8 NO-mediated angiogenesis is abrogated in β-catenin^−/−^ mouse pulmonary endothelial cells

**Figure 6:**
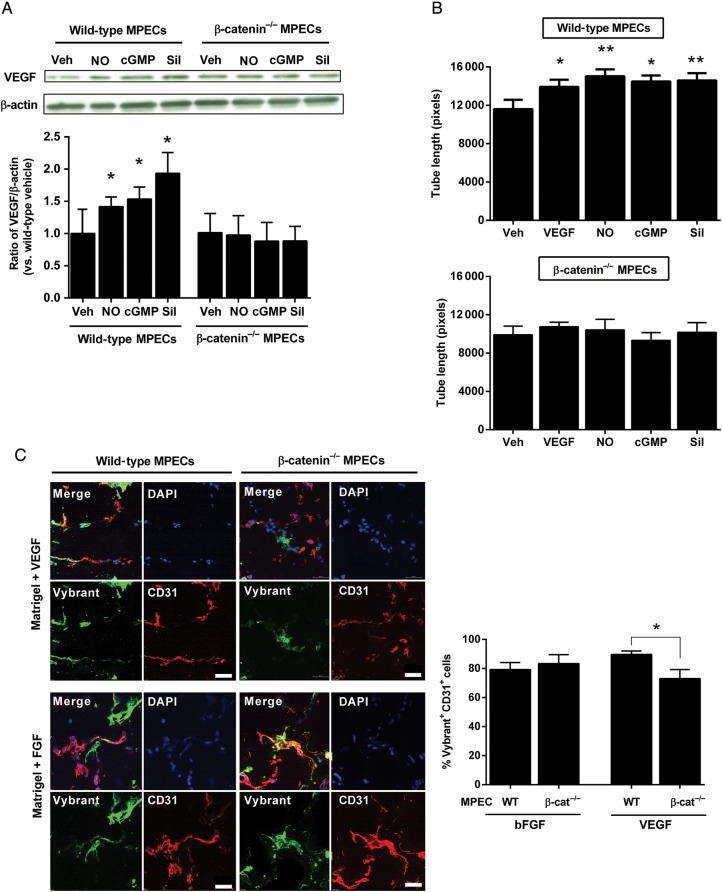
Loss of β-catenin impairs NO-cGMP-mediated migration and angiogenesis of mouse pulmonary endothelial cells. (*A*) Wild-type and β-catenin^−/−^ MPECs were treated with spermine NONOate (NO; 10 μmol/L), 8-bromo-cGMP (cGMP; 10 μmol/L) or sildenafil (Sil; 100 nmol/L) or vehicle (Veh) for 12 h, following which cell lysates were probed by western blotting for VEGF and results expressed as the densitometric ratio of VEGF to β-actin (shown relative to wild-type vehicle; *P < 0.05 vs*.* vehicle, one-way ANOVA with repeated measures; *n* = 4 per group). (*B*) Following treatment with these same agonists or with VEGF (50 ng/mL), wild-type and β-catenin^−/−^ MPECs were seeded onto Matrigel and tube formation assessed after 6 h (*P < 0.05, **P < 0.01 vs*.* vehicle, one-way ANOVA without repeated measures; *n* = 6–8 per group). (*C*) Mice were injected with Matrigel supplemented with VEGF (upper image panels) or bFGF (lower image panels) containing either wild-type or β-catenin^−/−^ MPECs labelled with Vybrant dye (green). Matrigel plugs were excised after 7 days, cryosectioned and immunostained with antibodies against CD31 (red) and mounted in DAPI (blue) and imaged using a confocal microscope (scale bar shows 50 µm). Results are expressed as the percentage of CD31-positive Vybrant-labelled MPECs compared with the total number of CD31-positive cells within four randomly selected fields of view per plug (*n* = 4 in each group; *P < 0.05 vs*.* wild-type MPECs, unpaired *t*-test).

NO-cGMP signalling plays a pivotal role in VEGF-induced angiogenesis, acting as a downstream effector of VEGF,^28–30^ and also regulating VEGF expression.^31,32^ We hypothesized that, since NO and cGMP increase transcriptional activity of β-catenin, loss of β-catenin may impair the NO-mediated angiogenic response. We assessed the expression of VEGF in wild-type and β-catenin^−/−^ MPECs following stimulation for 12 h with spermine NONOate, 8-bromo-cGMP or sildenafil, by western blotting of cell lysates. All three agents increased expression of VEGF compared with vehicle in wild-type, but not β-catenin^−/−^, MPECs (*Figure 6A*). A scratch-wound assay was performed to ascertain the migratory capacity of wild-type and β-catenin^−/−^ MPECs in response to stimulation (as discussed earlier), as well as to VEGF. Wound closure was increased by all agents in wild-type MPECs relative to vehicle, but not in β-catenin^−/−^ MPECs (Supplementary material online, *Figure S4A*).

Angiogenesis (tube formation) was assessed *in vitro* by seeding MPECs onto Matrigel following stimulation with VEGF, spermine NONOate, 8-bromo-cGMP, or sildenafil. Tube length was increased by all agents in wild-type, but not β-catenin^−/−^, MPECs relative to vehicle (*Figure 6B*). On the other hand both wild-type and β-catenin^−/−^ MPECs exhibited increased tube length following stimulation with bFGF (Supplementary material online, *Figure S4B*), an agent which stimulates angiogenesis independently of NO-cGMP signalling.^30^

To determine whether loss of β-catenin affects NO-mediated angiogenesis *in vivo*, a Matrigel plug assay was performed to assess capillary formation in plugs containing fluorescent-labelled wild-type or β-catenin^−/−^ MPECs in the presence of either VEGF or bFGF. In plugs supplemented with bFGF, capillary formation (assessed by the number of Vybrant-positive cells staining positive for CD31 as a percentage of total CD31-positive cells to take into account cells migrated from outside the plug) was similar between wild-type and β-catenin^−/−^ MPECs; however, in VEGF-supplemented plugs, β-catenin^−/−^ MPECs exhibited less Vybrant-positive CD31-positive cells and lower numbers of capillary-like structures or vascular networks compared with wild-type MPECs (*Figure 6C*). In parallel experiments, Matrigel was injected containing VEGF and either scrambled or β-catenin siRNA. The efficacy of β-catenin siRNA to reduce β-catenin protein levels was initially confirmed by western blotting of lysates following transfection of wild-type MPECs. Plugs containing β-catenin siRNA exhibited fewer CD31-positive cells per plug (indicating fewer endothelial cells migrated into the plug from surrounding tissue) compared with those containing non-targeting sequences (Supplementary material online, *Figure S5*). These data suggest that angiogenesis, driven by NO signalling, is attenuated following loss or knockdown of β-catenin.

## 4. Discussion

We here provide the first evidence that eNOS associates with β-catenin in endothelial cells, and that this association serves as a negative regulator of eNOS activity such that, when β-catenin is absent, basal eNOS activity/Ser1177 phosphorylation is increased and is unable to increase further in response to standard agonists (adenosine and histamine). We also show for the first time that NO, derived either from eNOS or exogenously, induces β-catenin translocation to the nucleus with resultant effects on gene transcription and endothelial function, in particular with regard to angiogenesis.

*In situ* proximity ligation assay revealed a pattern of co-localization of eNOS and β-catenin predominantly close to cell borders, which may be consistent with localization to caveolae. Both proteins are known to associate with caveolin-1^33–35^ and this newly identified interaction between eNOS and β-catenin may indicate a novel signalling complex. Caveolin-1 is a negative regulator of eNOS,^36^ and it is possible that the inhibitory action of β-catenin on eNOS function may be mediated through co-interaction with caveolin-1. This possibility deserves further investigation in future work. Analysis of proximity ligation assays also revealed that pharmacological activation of eNOS disrupts the interaction with inhibitory β-catenin, thus permitting NO production. This observation supports the concept that eNOS is subject to allosteric regulation by β-catenin, and also warrants further study in the future.

Our demonstration that NO-cGMP signalling induces nuclear translocation of β-catenin as well as downstream gene transcriptional effects supports previous work revealing induction of β-catenin signalling following activation of iNOS.^6^ Conversely, it has previously been shown in colon carcinoma cells, where β-catenin is constitutively active, that application of NO-acetylsalicylic acid reduces β-catenin transcriptional activity due to an inhibitory action of NO on the interaction between β-catenin and TCF-4.^37^ This suggests that in neoplastic cells, the communication between NO and β-catenin differs in comparison to contact-inhibited quiescent endothelial cells, where β-catenin expression and localization is highly regulated.

**Figure 7:**
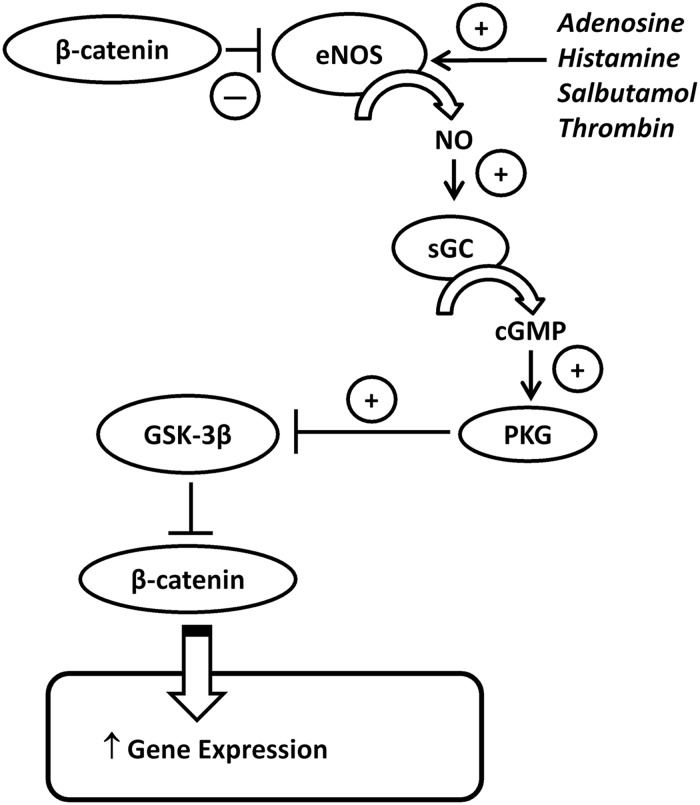
Schematic diagram of NO-β-catenin cross-regulation. In unstimulated cells, β-catenin is excluded from the nucleus due to the inhibitory action of GSK-3β (which phosphorylates β-catenin targeting it for degradation), while membrane-associated β-catenin binds to and inhibits eNOS activity. Following activation of eNOS using agonists, i.e. adenosine, histamine, salbutamol or thrombin, this association with β-catenin is lost. At the same time, agonists increase eNOS activity and NO production which increases the activity of soluble guanylate cyclase leading to increased production of cGMP which consequently increases the activity of PKG. PKG phosphorylates and inhibits GSK-3β which permits activation of β-catenin. Active (dephosphorylated) β-catenin is able to translocate to the nucleus where it exerts effects on gene transcription. The effects of eNOS activation are indicated by plus or minus symbols.

NO has previously been shown to directly *S*-nitrosylate β-catenin.^8^ Although we found that application of an exogenous NO donor mimicked the effect of eNOS activation on β-catenin nuclear translocation, which might be consistent with such a mechanism, we also found that inhibition of sGC (with ODQ) prevented this translocation in response to salbutamol; moreover, elevation of cGMP (with 8-bromo-cGMP or sildenafil) promoted nuclear translocation of β-catenin. These data suggest that the effect of eNOS activation or exogenous NO on β-catenin translocation is mediated through cGMP elevation rather than through direct NO-mediated *S*-nitrosylation of β-catenin, or indeed translocation of β-catenin released from active eNOS. cGMP exerts its intracellular signalling through activation of PKG. PKG has previously been reported to phosphorylate GSK-3β inhibiting its activity.^25^ We here show that activation of eNOS or exogenous application of spermine NONOate, increases phosphorylation of GSK-3β and that this is attenuated following inhibition of sGC or PKG. Although the PKG inhibitor may have non-specific effects at higher concentrations, the concentration used in this study is highly selective for PKG.^38^ Our results are therefore consistent with NO, derived either from eNOS or from an exogenously applied NO donor, leading to sGC/cGMP/PKG activation with resultant GSK-3β inhibition and thereby activation and stabilization of β-catenin through a decrease in its phosphorylation (summarized in *Figure 7*).

Many Ca^2+^-elevating eNOS agonists, such as histamine and thrombin, increase vascular permeability through disruption of the VE-cadherin/β-catenin complex.^39,40^ A recent study reported that exposure of microvascular endothelial cells to histamine resulted in increased levels of β-catenin in the nucleus.^41^ Similarly, stimulation with thrombin has also been found to promote nuclear translocation of β-catenin with consequent changes in gene expression.^42^ Consistent with these findings, our study has shown that histamine and thrombin induce β-catenin nuclear translocation, apparently through increasing intracellular Ca^2+^. However, this occurs independently of any effects of histamine and thrombin on eNOS activity or NO generation, since L-NAME co-incubation did not alter the effect of histamine or thrombin (unlike that of adenosine or salbutamol) on β-catenin nuclear translocation. It is possible that the nuclear translocation of β-catenin seen with these agents occurs secondary to nuclear translocation of associated eNOS. However, translocation is more likely to occur from cytosolic β-catenin (due to an increase in stabilization as a consequence of inhibition of the destruction complex) rather than plasmalemmal (and hence eNOS-associated) β-catenin, since we found no obvious changes in plasmalemmal distribution of β-catenin following 2.5 min treatment, although relocation from this compartment cannot be ruled out.

On the other hand, NO-mediated translocation of β-catenin to the nucleus does appear to result in transcriptional activation. Our data demonstrate that activation of NO-cGMP signalling using either an NO donor (spermine NONOate) or sildenafil, which inhibits cGMP phosphodiesterases and thereby elevates cGMP, results in the expression of β-catenin/TCF target genes, namely IL-8 and cyclin D1, which are important in regulating angiogenesis and proliferation. NO is well documented to modulate gene regulation at the transcriptional level in endothelial cells.^43–45^ This has been described as occurring through cGMP-mediated activation of transcription factors [in particular activating protein-1 (AP-1), cyclic AMP responsive element binding protein and early growth response protein-1] and through *S*-nitrosylation of transcription factors (in particular NF-κB, hypoxia-inducible factor-1, AP-1, nuclear factor-erythroid 2 p45-related factor 2, zinc finger transcription factors and heterogeneous nuclear ribonucleoproteins), as reviewed recently.^46^ Our data define a novel hitherto unrecognized mechanism by which NO regulates gene expression, namely through cGMP-dependent β-catenin nuclear translocation.

NO-cGMP signalling plays a key role in VEGF-induced angiogenesis,^28–30^ and it has been shown previously that angiogenesis and wound healing are impaired in eNOS-deficient mice.^47^ We provide evidence here that β-catenin is also required for NO-driven angiogenesis. Endothelial cells lacking the β-catenin gene exhibited reduced expression of VEGF in response to exogenously applied NO or elevation of cGMP. These cells also exhibited attenuated angiogenic and migratory responses to exogenous NO or to increased cGMP, as well as to VEGF (which requires eNOS/NO signalling). On the other hand, angiogenic responses to the NO-independent pro-angiogenic stimulus bFGF^30^ were intact in these cells. *In vivo* capillary formation by β-catenin^−/−^ MPECs was also attenuated in VEGF-supplemented Matrigel plugs when compared with capillary formation by wild-type MPECs, while there was no difference between these two cell types in Matrigel supplemented with bFGF. These data support the observation that β-catenin is required to mediate the pro-angiogenic effects of NO-cGMP signalling.

In conclusion, we have found in endothelial cells that β-catenin associates with eNOS under basal conditions, and that eNOS activation leads to β-catenin translocation to the nucleus through a cGMP-dependent mechanism with resultant effects on gene transcription and downstream functional responses, specifically as regards angiogenesis. We have also shown that β-catenin acts as an endogenous negative regulator of eNOS. Our findings provide novel evidence of bidirectional cross-talk and regulation between the NO-cGMP and the β-catenin signalling systems.

## Supplementary material

Supplementary Material is available at *Cardiovascular Research* online.

## Funding

This work was supported by grants from the China Scholarship Council (to N.C.), the British Heart Foundation (grant numbers PG/06/068/21100, PG/12/16/29443) and Wellcome Trust (WT087776MA).
